# Experiences of sleep problems, subsequent daytime consequences, and self-care activities used to improve sleep among patients with restless legs syndrome: a qualitative content analysis

**DOI:** 10.1177/17449871251384535

**Published:** 2025-11-11

**Authors:** Elzana Odzakovic, Annelie Ingelsbo Petersson, Emilia Lindholm Ericsson, Sandra Öberg, Malin Jakobsson, Maria Björk, Susanne Knutsson, Bengt Fridlund, Lise-Lotte Jonasson, Martin Ulander, Jonas Lind, Anders Broström

**Affiliations:** School of Health and Welfare, Jönköping University, Jönköping, Sweden; School of Health and Welfare, Jönköping University, Jönköping, Sweden; School of Health and Welfare, Jönköping University, Jönköping, Sweden; School of Health and Welfare, Jönköping University, Jönköping, Sweden; School of Health and Welfare, Jönköping University, Jönköping, Sweden; School of Health and Welfare, Jönköping University, Jönköping, Sweden; Department of Health and Caring Sciences, Faculty of Health and Life Sciences, Linnaeus University, Växjö, Sweden; Centre for Interprofessional Collaboration within Emergency Care (CICE), Linnaeus University, Växjö, Sweden; Centre for Interprofessional Collaboration within Emergency Care (CICE), Linnaeus University, Växjö, Sweden; School of Health and Welfare, Jönköping University, Jönköping, Sweden; Department of Clinical Neurophysiology, Linköping University Hospital, Linköping, Sweden; Department of Biomedical and Clinical Sciences, Division of Neurobiology, Linköping University, Linköping, Sweden; Department of Biomedical and Clinical Sciences, Division of Neurobiology. Linköping University, Linköping, Sweden; Section of Neurology, Department of Internal Medicine, County Hospital Ryhov, Jönköping, Sweden; School of Health and Welfare, Jönköping University, Jönköping, Sweden; Department of Clinical Neurophysiology, Linköping University Hospital, Linköping, Sweden; Department of Health and Caring Sciences, Western Norway University of Applied Sciences, Bergen, Vestlandet, Norway

**Keywords:** behaviour, nursing, life situation, qualitative content analysis, sleep hygiene, Willis Ekbom Disease

## Abstract

**Background:**

Restless legs syndrome (RLS) is a prevalent neurological condition affecting daily life. Symptoms can vary and worsen during the evening and night, with sleep problems as a common consequence. Few, if any, qualitative studies have explored how patients with RLS experience their sleep problems.

**Aim:**

The aim was to explore and describe how patients with RLS experience their sleep problems, the subsequent daytime consequences, and self-care activities used to improve sleep.

**Methods:**

An inductive, descriptive, qualitative design was used, including semi-structured interviews with 28 strategically selected patients from a national RLS organisation. Data were analysed with manifest qualitative content analysis and reported according to the COREQ checklist.

**Results:**

RLS-related ailments affecting sleep were: noticing initial symptoms in the evening, enduring stressful RLS symptoms at night, and being concerned about not having symptom-relieving treatment. Struggles with daytime consequences of poor sleep were: feeling excessive fatigue and managing social interactions. Self-care actions to improve sleep included trust in daily routines, benefiting from the use of various distractions, and actively seeking effective medical treatment.

**Conclusions and contribution to nursing:**

Knowledge about various RLS-related ailments affecting sleep can be used by nurses to provide adequate education about the disease and potential nursing interventions to improve sleep.

## Introduction

Restless legs syndrome (RLS) is a prevalent neurological sensorimotor disorder characterised by symptoms such as uncomfortable feelings and pain in the legs, arms, and sometimes other body parts (Gossard et al., 2021). To obtain the diagnosis of RLS, five clinical criteria described by Allen et al. (2014) must be met (Table 1). Motor restlessness, a need to move due to unpleasant sensations in the legs, and worsening symptoms at rest, are typical signs that occur or worsen in the evening and at night. Therefore, during clinical assessments, sleep problems are described as a common consequence (Khachatryan et al., 2022). The variation in symptoms and disease severity between patients generally, but notably between the sexes, is significant (Holzknecht et al., 2020). For example, sensory symptoms are more common in women, whereas motor symptoms are more common in men (Holzknecht et al., 2020). These variances lead to different sleep problems and lower quality of life compared to control groups (Broström et al., 2024). A few studies have reported how patients describe their RLS symptoms (Holzknecht et al., 2022), and how RLS affects the life situation (Harrison et al., 2021) and the fulfilment of human needs (Odzakovic et al., 2024a), but none of these studies have focused on how the actual sleep situation is experienced.

**Table 1.** Five clinical criteria that form the basis of restless legs syndrome diagnosis (Allen et al. 2014).

Need to move the legs and/or arms, often accompanied by discomfort and pain.
The need to move begins or worsens with rest and/or inactivity.
The need to move is alleviated, in whole or in part, immediately when you get up and walk.
The need to move and the discomfort worsen during evenings and nights.
The aforementioned complaints cannot be explained by other causes such as oedema, cramp, or inappropriate lying or sitting position.

According to a recent systematic review and meta-analysis, the worldwide prevalence of RLS is 3% (Broström et al., 2023). The risk of becoming affected increases with age (Khachatryan et al., 2022), although young adults and children can have RLS. Moreover, RLS is a common complaint during pregnancy (Tuna Oran et al., 2021). Genetic causes of RLS have been described (Khachatryan et al., 2022), and iron deficiency in the brain may contribute to its development (Ferré et al., 2019). Secondary RLS can stem from underlying diseases such as kidney failure or deficiency diseases such as B12 and folate deficiencies (Liu et al., 2022). The choice of treatment is guided by the severity of symptoms (Mackie and Winkelman et al., 2015). Several drugs are available, for example, dopamine agonists, L-dopa, alpha-2-delta ligands, opioids or iron, depending on the severity of symptoms. In the case of worsening symptoms, iron levels, ferritin and transferrin saturation should be checked along with the medical history (Gossard et al., 2021; Silber et al., 2021; Winkelmann et al., 2025). For some, non-pharmacological treatment (e.g. regular physical exercise, stretching, acupuncture, acupressure, cognitive behavioural therapy (CBT), cooling of the legs, and various types of mental distraction) may be sufficient (Harrison et al., 2019). Recently, a validated instrument to measure self-care behaviours among patients with RLS has been developed (Odzakovic et al., 2024b), but further studies on the frequency and benefits of the included behaviours are needed.

On polysomnography recordings higher percentages of N1 (i.e. light sleep, brief transitional phase from wakefulness) sleep and lower percentages of N3 (i.e. deep sleep which occurs mostly during the first part of the night, where the body performs vital restoration and growth, and it is very difficult to wake someone) sleep have been reported in men (Holzknecht et al., 2022), which might be explained by higher number of periodic leg movements during sleep (Shin et al., 2016). Moreover, two recent meta-analyses have described increased sleep latency (i.e. time it takes for a person to transition from full wakefulness to the onset of sleep after going to bed), shorter total sleep time, increased sleep fragmentation and lower sleep efficiency in patients with RLS compared to healthy controls (Geng et al., 2022; Zhang et al., 2022). Questionnaire-based quantitative studies have also shown that insomnia symptoms and daytime sleepiness are more common in those with severe RLS symptoms than in those with milder symptoms (Broman et al., 2008; Enomoto et al., 2006). Furthermore, poor sleep is a common reason that patients with RLS seek medical attention (Holzknecht et al., 2020). It is well known, however, that objective and subjective measures of sleep quality are only weakly correlated (Kaplan et al., 2017), and sleep questionnaires typically do not target the main symptoms of RLS (Fulda et al., 2021). To better assess the sleep situation in patients with RLS and to provide better advice regarding sleep problems, it is essential to understand how the patients themselves experience their sleep situation, including the consequences of poor sleep and how activities, including self-care during the day and evening, might impact sleep. To this end, qualitative studies are needed, and results from such studies could improve the understanding of the sleep situation to increase support for patients in their daily lives. However, only a few qualitative studies have been performed on patients with RLS (Harrison et al., 2021; Odzakovic et al., 2024a), and none of them, as far as we know, have specifically explored the sleep situation, daytime consequences of poor sleep, and self-care activities used to improve sleep. Therefore, this study aimed to explore and describe how patients with RLS experience their sleep problems, the subsequent daytime consequences, and self-care activities used to improve sleep.

## Methodology

### Study design and setting

An inductive, descriptive, qualitative design using manifest qualitative content analysis (QCA) (Graneheim and Lundman, 2004; Lindgren et al., 2020) was employed. The COREQ checklist was applied throughout the study to ensure comprehensive and transparent reporting of the qualitative methods, including participant selection, data collection, and analysis (Tong et al., 2007).

### Participants

A strategic selection (Graneheim and Lundman, 2004; Lindgren et al., 2020) was performed to achieve a clinically sound variation regarding gender, age, education level, cohabitation, comorbidity, and pharmacological treatment. A strategic sampling was utilised to ensure clinical diversity within the study population (Robinson, 2014). These criteria were shaped by the interdisciplinary research team’s theoretical and clinical expertise regarding the diverse manifestations of RLS across different contexts and disease trajectories. This approach aimed to capture a wide range of clinically relevant experiences and self-care practices among patients with RLS. An invitation letter to participate in a questionnaire-based interview was sent out to all members of the Swedish RLS Patient Association with the following inclusion criteria: age over 18 years, having been diagnosed and treated for RLS, ability to speak and understand the Swedish language, and ability to provide written informed consent. In total, about 1500 letters were sent. Of those, 788 agreed to participate. The questionnaire-based study has been described elsewhere (Hellström et al., 2024). All 788 participants were asked if they would agree to be interviewed, and 472 agreed. Background information used for strategic sampling was available from the questionnaires. Based on this, a strategic selection of 28 participants, chosen to ensure maximum variance regarding gender, age, educational level and civil status were approached and formally asked about participation in the present study. All 28 agreed.

### Data collection

A written information letter about the qualitative interview study was sent out to the 28 strategically selected participants, who all agreed to participate. The interdisciplinary research team (i.e. physicians, nurses, and sociologists), who had extensive competence regarding the treatment of patients with RLS and doing studies with qualitative content analysis, developed and pilot tested a semi-structured interview guide including open (e.g. would you please share your experiences of what a typical night might look for you?) and probing questions (e.g. can you provide further explanation regarding the occurrence and experiences of nocturnal symptoms and how they affect your sleep?) about their sleep situation (Lincoln and Guba, 1985). No changes were made to the semi-structured interview guide after the pilot interview. Clarification probing questions were employed to ensure a comprehensive exploration and to confirm our grasp of the collected information. The interviews were conducted via telephone during June and November 2022. After obtaining informed written consent, the researcher provided additional information about the interview and answered participant-initiated questions regarding the study. Fieldnotes were not taken during the interviews. Each participant was interviewed once because of the depth of the interview data and its duration. To meet the unique needs and experiences of our participants, we customised the telephone interviews accordingly (Novick, 2008). The interviews were audio-recorded and planned to last between 45 and 90 minutes. The participants had the option to request shorter sessions or to split them into multiple parts. We scheduled the interviews to align with the participants’ daily routines and symptom patterns, incorporating regular check-ins and breaks to ensure their comfort and promote engagement throughout the process.

### Data analysis

Verbatim transcripts of all interviews were produced, resulting in an analysis unit. A manifest analysis, based on the Graneheim and Lundman model (Graneheim and Lundman, 2004) for qualitative content analaysis, was used. By employing a manifest level of analysis, we gradually reached a deeper understanding of the phenomenon being studied and concentrated on the observable content, emphasising what the text explicitly stated (Graneheim and Lundman, 2004). The interview transcripts were not returned to the participants. Initially, the interviews were read and discussed by all authors several times to identify meaning units based on the current aim. In the next step, the meaning units were read and discussed by all authors repeatedly and then compared. Then, the meaning units were condensed into smaller units, a process where the text was shortened while ensuring the core content was preserved. Based on the meaning units, several codes were created to briefly describe the textual content, and these were then compared for similarities and differences. Furthermore, the codes with similar content were inductively sorted into subcategories. All subcategories with corresponding meanings were finally grouped into different categories. All subcategories and categories addressed the aim of this study and ultimately constituted the manifest content and thus the study’s results. The research team regularly discussed codes, subcategories and categories, resolving discrepancies through dialogue. Agreement was reached collaboratively at each stage, ensuring a reliable and valid category system. Finally, the interdisciplinary research team engaged in discussions to establish a consensus and to validate a category system describing how patients with RLS experienced their sleep situation. To ensure the rigour of the study, we addressed all four criteria of trustworthiness by Lincoln and Guba (1985). We achieved credibility through in-depth engagement with the data and by triangulating among authors and patients with RLS. Transferability was supported by providing rich, detailed descriptions of the study context and participants. We maintained dependability through a thorough audit trail that documented all research decisions and analytical procedures. Lastly, we enhanced confirmability by keeping reflexive notes and involving multiple authors in the coding and interpretation process, ensuring that the findings reflected the participants’ experiences rather than any researcher bias.

### Ethical considerations

Ethical approval for this study was granted by the Swedish Ethical Review Authority (reference: 2022-01515-01). The research was conducted in line with the Declaration of Helsinki (World Medical Association, 2024). All participants received both written and verbal information about the study, and written informed consent was obtained. At the beginning of each interview, the interviewer reiterated the study information and the participants’ rights, emphasising that participation was voluntary, and they could withdraw at any time without providing a reason. Given that patients with RLS may be considered a vulnerable group in research, special attention was paid to their comfort and well-being during the interviews. The interviewer monitored participants’ reactions while discussing their experiences and was available for follow-up conversations if needed; however, no participants required this. The primary ethical considerations included voluntary participation, informed consent, confidentiality, and secure data handling. Invitation letters and participant information sheets were carefully crafted to address these issues. Personal data were anonymised using unique identification numbers and stored securely in an encrypted file, accessible only to the research team. All data utilised anonymised identifiers, and data storage and handling complied fully with GDPR standards. This study was considered low-risk, with small likelihood of causing distress. All members of the interdisciplinary research team have professional medical and nursing backgrounds and extensive experience in conducting interviews, which ensured ethical sensitivity and participant safety throughout the study.

## Results

The characteristics of the sample are described in Table 2. The three categories were: *suffering from RLS-related ailments affecting sleep, struggling with daytime consequences of poor sleep*, and *using self-care actions to improve sleep.* Each category has three subcategories (Table 3). Quotations are presented to illustrate the categories and subcategories.

**Table 2.** Demographics and clinical characteristics of patients with restless legs syndrome (*N* = 28) interviewed in the study.

Variables	Value
Gender, female, *n* (%)	16 (57)
Age (years), mean (range)	67.6 (39–89)
Educational level, *n* (%)	
9 years or below	3 (11)
12–13 years	11 (39)
University	14 (50)
Civil status, *n* (%)	
Married/living together	20 (71)
Unmarried and living alone	3 (11)
Divorced/widower and living alone	5 (18)
Smoking, *n* (%)	
Yes, *n* (%)	1 (4)
Alcohol, *n* (%)	
Never uses alcohol	8 (29)
Uses alcohol at least once a month	9 (32)
Uses alcohol 2–3 times or more/week	11 (39)
Comorbidity, *n* (%)	
Renal disease	0 (0)
Parkinson’s disease	0 (0)
Multiple sclerosis	0 (0)
Migraine	3 (11)
Iron deficiency	4 (14)
Pharmacological treatment, *n* (%)	
Dopamine agonists	22 (80)
Opioids	8 (29)
α2δ Ligands	7 (25)
Dopa/derivates	5 (20)
Iron supplement	3 (11)

**Table 3.** Categories and subcategories according to Graneheim and Lundman model (2004) describing the sleep problems among patients with restless legs syndrome (*N* = 28).

Quotations	Subcategories	Categories
‘As soon as I sit down in the evening, tired, I can’t sit still. I have to walk back and forth’. (Man 65 years old) ‘I wake up after 2 hours and then must get up and walk around. I must get up! I must do something because it’s impossible to lie in bed. And in the worst case, it might happen that I can't fall asleep again until 4 a.m. and then, maybe, get an hour of sleep’. (Woman 79 years) ‘At the primary care center, they prescribed sleeping pills and all sorts of things that only made it worse’. (Woman 68 years old)	*Noticing initial symptoms in the evening* *Enduring stressful RLS symptoms at night* *Being concerned about not having a symptom-relieving treatment*	**Suffering from RLS-related ailments affecting sleep**
‘I’ve probably never woken up refreshed and well-rested, so I miss that. I miss waking one morning and feeling, ‘Wow, now I'm going to seize the day or be able to do things energetically’, but I’m exhausted, and the days are long’. (Woman 35 years old) ‘It feels like psychological torture. I have underestimated the effect, I must say, the lack of sleep and how it affects one’s psyche’. (Man 54 years old) ‘If I've had a lot of crawling at night, I can be very tired. But they know about this at work, so if it’s been a bad night and I don’t have anything urgent, I can call in sick’. (Woman 51 years old)	*Feeling excessive fatigue* *Feeling mental distress* *Managing social interactions*	**Struggling with daytime consequences of poor sleep**
‘If I forget the pills, then my sleep is ruined. So, it is important to remember’. (Man 60 years old) ‘I try to move and imagine that it might be good. Because it gets worse when I relax and rest and try to sleep like that’ (Woman 80 years old) ‘I want to know what ongoing research and new medications are available. I have tried one or two patches, but it’s on my own initiative, my request; no doctor is addressing my situation’. (Women 62 years old)	*Trusting in daily routines* *Benefiting from a variety of distractions strategies* *Actively seeking effective medical treatment*	**Using self-care actions to improve sleep**

### Category 1: Suffering from RLS-related ailments affecting sleep

RLS-related symptoms were experienced at different times of the day, but those appearing early in the evening caused severe stress as they often led to a bad night’s sleep. Throughout the night, the patients struggled with the challenging symptoms; meanwhile during the day, worries about the adequacy of their treatment and its ability to relieve symptoms were mentioned.

#### Noticing initial symptoms in the evening

Significant stress and fear were expressed due to feeling RLS-related symptoms, especially if they started early in the evening, or seemed more intense than before. The stress itself was also perceived as a trigger that can increase the severity of symptoms. Typical symptoms such as pain and a crawling feeling in the legs and upper extremities were common, as well as leg cramps, involuntary leg jerks and difficulty sitting still. The intensity and duration tended to worsen the longer the patient had lived with RLS. Even though mild transient symptoms were more common among those with a recently diagnosed RLS, severe persistent symptoms were also present. The unpredictability was another stressor, described as not knowing when a period with absence of, or mild, symptoms could change into weeks with severe symptoms.

#### Enduring stressful RLS symptoms at night

Extensive sleep problems, including difficulty falling asleep in the evening, or waking up during the night and struggling to return to sleep again, were described. A crawling sensation in the legs along with pain was familiar, as well as tense muscles, leg twitching, or a general discomfort in the body. There was a discrepancy in when the symptoms occurred, for example, in the evening before going to sleep, at sleep onset, or after 4–5 hours of sleep. The duration varied greatly, which meant wakefulness could last from one to several consecutive hours. Severe persistent symptoms were described as a cause of being awake most of the night. When the symptoms had diminished during the early morning hours, it was possible to get some hours’ sleep. The duration of morning sleep was linked to employment status, sick leave, or retirement, and those who had the opportunity sometimes slept until 10–11 in the morning.

#### Being concerned about not having symptom-relieving treatment

A lack of effective pharmaceutical treatment caused concerns and brooding, which disturbed sleep. Some prescribed drugs did not reduce RLS symptoms or sleep problems, while others, such as antipsychotics, even tended to worsen symptoms. Additionally, the efficacy of initially effective drugs seemed to diminish over time. Other solutions were therefore tried. A change in dosage, for example, executed as an increased intake of RLS medications on the patient’s own initiative, based on a belief that it would reduce symptoms and improve sleep quality, was expressed, and higher doses than those prescribed by the physicians were used. Concerns regarding physicians’ attention to symptoms and potential treatment side effects were also described, often resulting in challenges when discussing adjustments to medication regimens. Anxiety about forgetting to take the proper medication at the appropriate time, and if so, disrupting sleep the following night, was also expressed. Others feared becoming overmedicated or addicted.

### Category 2: Struggling with daytime consequences of poor sleep

The RLS-related sleep deprivation and subsequent experience of excessive fatigue were described as significantly impacting psychological well-being and causing feelings of mental distress, which created challenges in social interactions.

#### Feeling excessive fatigue

Excessive fatigue during daytime was described as a common problem. This was manifested in physical symptoms such as frequent headaches, exhaustion and sleepiness, which sometimes meant unintentionally nodding off, the latter being particularly stressful if it occurred in inconvenient social- or work-related situations. Having family and friends commenting on a tired appearance added strain. The fatigue also caused negative emotional consequences, with feelings of listlessness being emphasised.

#### Feeling mental distress

Lack of sleep was described as a cause of mental distress, which in turn negatively affected various aspects of the life situation. Anxiety, fuelled by fear of long-term health consequences associated with poor sleep, was common. Sadness and low mood, also described as depressive symptoms, occasionally requiring treatment with antidepressants, were mentioned by others. Negative cognitive consequences, such as difficulties managing creative thinking, maintaining concentration when doing intellectual tasks and difficulties remembering things in general, caused mental distress. Feelings of irritability and anger were other problems, which decreased patience in social interactions.

#### Managing social interactions

There were various difficulties in social interactions with family, friends, and colleagues at work. When RLS symptoms were troublesome, it often required adaptation to avoid disturbing the partner’s sleep, which sometimes resulted in separate bedrooms. Having a partner who understood the sleep problems and how to manage them required open, constructive dialogue. Carrying out tasks at home, such as running errands or cleaning, was difficult due to tiredness and could create conflicts. Going to see a movie, a play or a concert was challenging, as were enjoying parties involving alcohol or travelling, especially during periods when pronounced RLS symptoms were present. However, the participants were aware of when symptoms started and ended and therefore often tried to make the most of social interactions during symptom-free periods. Those employed faced difficulties concentrating at work, sometimes prompting a search for alternative employment. A night of poor sleep increased the risk of arriving late at work, needing rest, taking a nap, or leaving early. A flexible, understanding attitude from management was described as beneficial. Those with milder symptoms reported a minimal impact on their work.

### Category 3: Using self-care actions to improve sleep

The importance of planning daytime and evening activities to improve sleep, alongside maintaining a calm bedtime environment was emphasised. Managing symptoms through distraction, relaxation techniques, and medical treatment, including prescribed RLS drugs and supplements, was common for symptom relief and improved sleep quality.

#### Trusting in daily routines

The importance of planning daytime and evening activities, as well as recognising their positive impact on sleep quality was emphasised. Establishing a bedtime schedule aimed at improving sleep helped alleviate nighttime symptoms. It was also important to create routines that resulted in a calm, peaceful, cool, and well-aired bedroom. Taking medications at the correct times was prioritised, with dossiers used to aid remembering, or having routines as contextual cues to ensure timely intake, particularly before bedtime. Heavy and late meals were believed to delay the effects of the medication. The disruptive effects of being out late at night were acknowledged, and activities were adapted accordingly to minimise negative impacts on sleep. Making up for lost sleep during daytime was described as a must by those with severe nighttime symptoms. Others expressed that adopting a leisurely pace and not sleeping too long during the day improved sleep during the night, when they had fewer symptoms.

#### Benefiting from a variety of distraction strategies

Solving crosswords, doing household tasks while standing, watching television programmes, reading books and newspapers, and listening to audiobooks, podcasts, or music to relax at night were described as distraction techniques when symptoms were less demanding. When symptoms were worse, physical activities such as walking, doing push-ups, jumping, stretching, pedalling an exercise bike, standing and rocking, or walking a few kilometres at night were symptom-relief strategies. Additionally, massage and pressure, applying cold or warm water to the legs, cooling the legs by standing on cold grass, or walking out in the snow in winter provided symptom relief. The choice of distraction techniques and symptom-relief strategies often depended on the severity of symptoms and the physical location. Having access to more space facilitated physical activities such as walking or stamping. There were also participants who, during the daytime, preferred lying on a sofa or sleeping in an upright position, while others avoided daytime rest to ensure better nighttime sleep quality.

#### Actively seeking effective medical treatment

Effective medical treatment was frequently sought to decrease symptoms and enhance sleep quality. Additional drugs, such as painkillers and sleeping pills, were consumed alongside prescribed RLS drugs to achieve better sleep despite occasional restlessness. Adhering to medication schedules, including exact time points for intake, was deemed crucial, as was caution regarding medication-diet interactions. Participants experienced varying effects from RLS-related drugs over time, prompting them to explore alternative options. Melatonin and supplements such as selenium, Vitamin D, and magnesium were used.

## Discussion

This study, which aimed to explore and describe how patients with RLS experienced their sleep problems, is, to the best of our knowledge, the first of its kind. The results describe a wide variety of RLS-related ailments that disturbed sleep, how sleep itself was affected, but also various physiological, psychological, cognitive, behavioural, and social daytime consequences of poor sleep. We also identified several different self-care actions that were used to improve the sleep situation.

Not surprisingly, the situation was perceived as complex, which aligns with studies objectively recording sleep and that describe increased sleep latency, shorter total sleep time, increased sleep fragmentation, and lower sleep efficiency (Geng et al., 2022; Zhang et al., 2022). Several factors, either indirectly or directly related to the disease, contributed to the sleep problems. To begin with, typical RLS symptoms, such as a crawling sensation in the legs and pain, tense muscles, leg twitching, or general discomfort in the body were frequently described as disturbing sleep. Difficulties falling asleep were common, as was awakening after a few hours of sleep, which led to periods of wakefulness, in many cases for several hours. The waking time was spent walking, stamping, or doing various self-care activities to cope with the symptoms. In the present study, stressful feelings caused by RLS symptoms during the evening or experiences of more severe symptoms could be seen as precipitating biological and psychological factors for insomnia. The occurrence of insomnia among patients with severe RLS symptoms has been documented in several questionnaire studies (Broman et al., 2008; Enomoto et al., 2006). Even if RLS symptoms were known to increase in the evening and at night, the patients in the present study expressed that when symptoms occurred early in the evening, they often caused fear and severe stress as they were perceived as a sign that a night with poor sleep would follow. The unpredictability of the situation, described as a symptom-free or stable period suddenly being disrupted by severe symptoms, created additional stress, which led to difficulties falling asleep. It is understandable that the feeling of severe RLS-related symptoms (Holzknecht et al., 2020, 2022) was described as unbearable and as generating peaks of stress (Romero-Peralta et al., 2020). Moreover, RLS is a fluctuating long-term condition (Khachatryan et al., 2022) which, when symptoms are severe, creates barriers to satisfying several human needs (e.g. physiological needs to rest and sleep, social needs to participate in social events with friends [Odzakovic et al., 2024a]). This can lead to psychological consequences (e.g. depressive symptoms [Chenini et al., 2022]). The perception of the symptom burden (Salas and Kwan, 2012) is an important risk factor to consider as regards the development of sleep problems over time.

The 3P Disease Model (Wright et al., 2019) describes the interaction between various factors and how a general sleep problem can evolve from a premorbid, or acute, into an early chronic, or chronic, insomnia phase. This model categorises these factors into three groups: predisposing, precipitating, and perpetuating. Predisposing factors create vulnerability to sleep issues, while precipitating factors trigger disturbances in sleep. Perpetuating factors, on the other hand, maintain the condition. This framework illustrates how temporary sleep problems can develop into chronic insomnia (Wright et al., 2019). Ellis et al. (2021) have tested the model and believe that an essential aspect of the development of chronic insomnia is the person’s vulnerability to stressors. Although initiation or adaptation of treatment, or mitigating use of self-care activities, as described by the patients in the present study, may decrease the RLS symptoms to an acceptable level, the precipitating factors might come back because RLS symptoms vary, and treatment optimisation is complex (Silber et al., 2021). The situation can be perpetuated by new or increased symptoms or psychological or socio-environmental consequences of previously experienced symptoms. Consequently, when new RLS symptoms are experienced, the stress level increases and sleep problems will return above the insomnia threshold, which can create a chronic insomnia situation over time (Perlis et al., 2011). The existence of stress, including biological, psychological, and social aspects, is comprehensive when suffering from RLS. In the present study, mental distress (i.e. anxiety and depressive symptoms) and difficulties in social interactions (i.e. with family, friends and colleagues at work) were described as psychological and social consequences that could worsen sleep problems. Notably, Chenini et al. (2022) showed that the frequency of depressive symptoms was ten times higher in chronic diseases and that depression was more common among patients with RLS than expected. Several studies not conducted on patients with RLS specifically (Ashworth et al., 2015; Manber et al., 2016; Norell-Clarke et al., 2018) have shown that the risk of developing depression increases the more stress the person is exposed to, and that insomnia can lead to depression. It is also stated that insomnia has a more significant impact on depression than depression has on insomnia. As described in the present study, RLS symptoms might act as a constant underlying stressor and will keep the patient close to or above the threshold for insomnia (Wright et al., 2019). Detecting sleep problems and mental symptoms at an early stage is of the utmost importance to prevent and treat them, thus increasing the patient’s well-being. Nurses should adopt a holistic approach, as our findings indicate that several factors contribute to a vicious cycle. In this cycle, symptoms lead to psychological reactions such as fear and stress, which then result in disturbed sleep, daytime consequences, and social limitations. To break this cycle, nurses must combine their professional competence with interpersonal skills, dedication, clarity of values, and self-awareness. This combination will enable them to provide comprehensive, patient-centred care for patients with RLS (McCance and McCormack, 2025). However, future studies using quantitative methods should explore the relationships among RLS symptoms, stress, depression, and sleep, as well as the mediating roles of depression and stress in the association between RLS symptoms and sleep quality. CBT is an established treatment for insomnia (Riemann et al., 2023). Song et al. (2020) showed in a small-scale study that face-to-face CBT is a potential method to treat sleep problems in patients with RLS since insomnia symptoms decreased when it was used.

In the present study, the lack of sleep was described as causing several consequences in daily life. Notably, there was a feeling of excessive fatigue, which caused physical symptoms such as headaches, but also listlessness, resulting in a low desire to do things during the day. Specific studies on the social consequences of RLS-related fatigue are not to be found, but a recent systematic review (Torossian and Jacelon, 2021) showed that even if patients with RLS were not the focus, noteworthy associations existed among fatigue, comorbidities, depression and anxiety, as well as lack of well-being. Negative correlations between self-care, sleepiness and fatigue were also described. Moreover, poor sleep, low physical activity, educational status, and socioeconomic status were other consequences to consider as factors contributing to increased feelings of exhaustion, which limited social capacity. Our patients described that participation in social contexts often meant sacrifices, especially regarding late-night activities, or those involving alcohol, as sleep became negatively affected. A recent study (Odzakovic et al., 2024a) focusing on facilitators and barriers to the fulfilment of human needs when living with RLS, described losses of meaningful friendships and anxiety about the loss of social capacity. Moreover, Harrison et al. (2021) reported that social and intimate relationships are affected by the inability to sit and relax with people. Unfortunately, no studies have specifically investigated the partner perspective on sleep in an RLS context. Still, it is important to acknowledge that, in general, lack of sleep affects relationships negatively, as bed partners are often affected (Odzakovic et al., 2024a). In the present study, the patients were aware that their bed partner and other family members can be disturbed when RLS symptoms occurred at night. They acknowledged that this created a need for separate bedrooms to avoid disturbing the partner. Therefore, it is important that partners are included in the care and, if accompanying a patient to a clinical appointment, are asked about their own insomnia symptoms, and receive adequate information and support (Say et al., 2020). Another aspect of daily life to consider is work. Being late to work in the morning, difficulties concentrating and staying awake, as well as a need to rest during the day, occurred during periods with severe RLS symptoms. A need to change workplace because of tiredness, which affected work performance, was not always possible and was perceived as poor flexibility by the employer. However, tiredness and excessive daytime sleepiness can be important risk factors and increase the risks of work-related accidents, which makes them important aspects to be discussed during clinical appointments (Uehli et al., 2014).

Interestingly, the patients described using a variety of self-care behaviours in different situations. The effectiveness of the self-care behaviour was perceived to depend on the severity of the symptoms. Harrison et al. (2019) suggest that self-care, either with pharmacological treatment or when pharmacological treatment is not sufficient, can help patients with RLS manage symptoms and daily life. Exactly what self-care stands for in an RLS context can be debated, since only one study has specifically investigated the patient’s perspective of self-care (Odzakovic et al., 2025). Self-care in relation to chronic conditions has been defined as the capacity to attend to one's well-being and health in everyday life through awareness, self-control and self-reliance for achieving, maintaining, or coping with the symptoms of a disease without continual support from a nurse (Jaarsma et al., 2017; Riegel et al., 2012). We found patients used sleep hygiene actions, including consistent routines and optimising their bedroom environment, alongside physical activities such as walking, stretching, or massage to manage evening and nighttime symptoms. They also employed distraction techniques, like listening to music or podcasts and doing mental tasks, to cope with sleep situation. Riegel et al. (2024) stress that self-care is essential in the management of a long-term illness and represents an active decision-making process that empowers individuals to effectively participate in their healthcare. Finding the right pharmacological treatment for RLS can be challenging (Allen et al., 2014), partly due to the risk that, despite an initial improvement in symptoms and sleep situation after taking medication, symptoms return and may worsen over time, so-called augmentation (Tachibana, 2015). Unfortunately, the cumulative impact of self-care interventions and pharmacological treatment, whether implemented individually or together, remains unclear due to limited research and the lack of disease-specific instruments in this area (Harrison et al., 2019). However, this knowledge gap can be filled since a recently developed and validated short (i.e. eight-item) disease-specific questionnaire, the RLS-Self-care Behaviour Questionnaire (RLS-ScBq), can be used to assess the use and benefit of self-care behaviours (Odzakovic et al., 2024b). Future studies using quantitative methods should examine the relationships between RLS symptoms, pharmacological treatment and the use and benefits of the self-care actions included in the RLS-ScBq among patients with various levels of disease severity. Specifically, the focus can also be on comparisons regarding how sociodemographic (e.g. age, gender, educational level and health literacy) and clinical factors (e.g. sleep quality, daytime sleepiness, depression and shared decision-making) differ between patients with high-versus-low use of self-care actions.

### Limitation

There are several limitations to consider. Firstly, the participants were derived from a patient organisation, which possibly impacted their knowledge about how to deal with the disease and its treatment, which in turn might have affected how the sleep situation was experienced. However, the sample included a total of 28 strategically selected participants, which was large for a qualitative study, and showed a great variation contributing to clinical credibility and transferability (Lincoln and Guba, 1985). Secondly, the data were collected through telephone interviews with three multi-professional interviewers without visual cues. Even though this created a relaxed environment for discussing sensitive information, the potential bias from the interviewers must be considered. This was addressed through extensive discussions among the research team, allowing different perspectives on the sleep situation to emerge, which enhanced dependability and confirmability. Thirdly, the telephone interviews offered flexibility and reduced travel burdens; however, the length of telephone interviews (45–90 minutes) could cause fatigue in some participants. To reduce discomfort or fatigue, breaks, shorter sessions and scheduling around symptom patterns were implemented. Fourthly, the aim of the study was to explore and describe how the patients experienced sleep problems, the subsequent daytime consequences and self-care activities used to improve sleep, which might have been a broad topic to describe. However, as RLS symptoms often occur in the afternoon and evening (Gossard et al., 2021), which affects the possibilities of preparing for going to bed and falling asleep, these aims might have helped the participants to provide rich, varied, and comprehensive descriptions of how daytime, evening, as well as nighttime aspects, including self-care activities, affected their sleep situation.

## Conclusion

Despite RLS being a well-known neurological disease, there is still a knowledge gap that needs to be filled in terms of how patients experience their sleep situation. Several factors, indirectly or directly related to the disease, contributed to various daytime consequences. Different self-care actions were used to relieve symptoms. Nurses’ ought to use knowledge about precipitating factors, consequences and self-care activities to offer adequate information about the disease and potential care interventions and to refer the patient to the right level of care. There is a great need for research on how self-care activities can constitute a treatment strategy, either with pharmacological treatment or when pharmacological treatment is insufficient.

**Key points for practice, policymaking and/or research**

- This study highlights the various symptoms of RLS that disrupt sleep and the wide-ranging daytime effects of poor sleep.
- Understanding patients’ self-care strategies allows nurses to incorporate them into care and education, promoting more effective self-management of RLS-related sleep problems.
- Nurses can utilise insights into symptom triggers, impacts, and self-care behaviours to guide patient education, tailor nursing interventions, and ensure appropriate referrals.
- Policies and clinical practice should support personalised RLS care by integrating symptom patterns, treatment effects and patients’ self-care strategies to improve outcomes and optimise healthcare resources.
- Healthcare providers in clinical practice should actively involve patients in developing individualised care plans, empowering them to manage their symptoms effectively and improve sleep quality and daytime functioning.
- Future randomised controlled trial studies with large sample sizes, implemented through the Internet to increase accessibility, could be conducted to establish the long-term effects of CBT on sleep problems (i.e. insomnia and daytime sleepiness), RLS symptoms and quality of life.

## Supplemental Material

sj-pdf-1-jrn-10.1177_17449871251384535 – Supplemental material for Experiences of sleep problems, subsequent daytime consequences, and self-care activities used to improve sleep among patients with restless legs syndrome: a qualitative content analysis Supplemental material, sj-pdf-1-jrn-10.1177_17449871251384535 for Experiences of sleep problems, subsequent daytime consequences, and self-care activities used to improve sleep among patients with restless legs syndrome: a qualitative content analysis by Elzana Odzakovic, Annelie Ingelsbo Petersson, Emilia Lindholm Ericsson, Sandra Öberg, Malin Jakobsson, Maria Björk, Susanne Knutsson, Bengt Fridlund, Lise-Lotte Jonasson, Martin Ulander, Jonas Lind and Anders Broström in Journal of Research in Nursing
